# Maternal outcomes among ostomy patients undergoing vaginal and cesarean deliveries: A national analysis

**DOI:** 10.1002/pmf2.70261

**Published:** 2026-02-12

**Authors:** Sona Mahrokhi, Amulya Vadlakonda, Troy Coaston, Gizem Bilgili, Melissa Justo, Konmal Ali, Yalda Afshar, Peyman Benharash

**Affiliations:** ^1^ Center for Advanced Surgical and Interventional Technology Department of Surgery David Geffen School of Medicine at UCLA Los Angeles California USA; ^2^ Department of Obstetrics and Gynecology David Geffen School of Medicine at UCLA Los Angeles California USA; ^3^ Department of Surgery David Geffen School of Medicine at UCLA Los Angeles California USA

**Keywords:** delivery, nationwide readmissions database, ostomy

## Abstract

**Background:**

With the increasing prevalence of conditions requiring stoma creation in younger populations, a growing number of women with ostomies are considering pregnancy. However, limited data exist regarding maternal outcomes at the national level.

**Methods:**

This retrospective cohort study analyzed the National Readmissions Database from 2016 to 2022. All adult hospitalizations involving vaginal or cesarean delivery were identified using *International Classification of Diseases 10th Revision* codes and stratified based on documentation of an ostomy diagnosis code during the index hospitalization. The primary outcome was severe maternal morbidity (SMM). Secondary outcomes included individual complication categories, in‐hospital mortality, and resource utilization. Risk‐adjusted analyses were performed using entropy balancing and multivariable regression models.

**Results:**

Among 13,292,764 delivery hospitalizations, 926 (∼0.01%) involved pregnant women with ostomies. The prevalence of ostomy during deliveries increased 1.5‐fold during the 7‐year study period from 2016 to 2022, from 7.1 to 10.6 per 100,000 deliveries overall, with specific increases observed for both cesarean (15.3 to 19.9 per 100,000) and vaginal (3.0 to 7.5 per 100,000) deliveries. After risk adjustment, ostomy was associated with increased odds of SMM (adjusted odds ratio [AOR], 1.57; 95% CI, 1.04–2.36). Treatment at medium (AOR, 0.95; 95% CI, 0.94–0.97) and high‐volume emergency general surgery (EGS) centers (AOR, 0.94; 95% CI, 0.92–0.96) was associated with reduced odds of SMM. Ostomy was linked with increased hospitalization costs by $1.18k (95% CI, 473–1890), and tendency toward higher odds of 30‐day readmission (AOR, 1.54; 95% CI, 0.91–2.60).

**Conclusion:**

Maternal ostomy is associated with increased SMM and resource utilization during birthing hospitalizations. However, specialized care at medium and high‐volume EGS centers may reduce these risks. These findings highlight the need for specialized protocols for pregnant women with ostomies to optimize maternal outcomes.

## 1 INTRODUCTION

An estimated 750,000 Americans live with an ostomy, while ∼130,000 operations involving the creation of an intestinal stoma are performed annually in the United States [1, 2]. While the management of stomas is complex both in the short and long term, they are an integral part of the treatment pathways for myriad pathologies including inflammatory bowel disease, colorectal malignancy, trauma, and other emergent abdominal pathologies. Trends in ostomy utilization vary by underlying indication. Although ostomy creation for inflammatory bowel disease may be declining in the era of highly effective biologic therapies, ostomies continue to be required in younger patients for other conditions, including colorectal cancer, which has demonstrated a rising incidence among reproductive‐age individuals [3, 4]. As a result, clinicians increasingly encounter patients with ostomies who are of reproductive age and may consider pregnancy [5, 6]. Given significant advances in the longitudinal management of ostomy and associated underlying conditions, women with ostomy are more frequently considering pregnancy [7]. However, pregnancy results in significant physical and physiological changes including increased intra‐abdominal pressure, expanding uterine size, and altered gastrointestinal motility, all of which may negatively influence stoma function. Historically, pregnancy in patients with an ostomy was approached with caution and often managed conservatively, reflecting limited available evidence and concerns regarding stoma‐related complications [7, 8, 9], with improved patient outcomes noted among centers utilizing multidisciplinary care coordination with maternal‐fetal medicine specialists [10].

Bouwknegt and coworkers demonstrated that pregnancy with a stoma is feasible, though associated with manageable complications including a 50% cesarean rate and significant stoma‐related issues such as obstruction and decreased output in 71% of cases [5]. A study by Kelly et al. reported high rates of cesarean birth and preterm delivery, with 31.25% of pregnancies experiencing stoma complications [6]. These studies collectively suggest that while pregnancy is possible for women with stomas, it is often accompanied by higher rates of surgical and obstetric complications. Nonetheless, available studies have been limited by sample size and a national perspective on the topic remains lacking.

In the present work, we characterized the association of maternal stoma with clinical and financial outcomes of birthing hospitalizations at the national level. We hypothesized ostomy pregnant patients to be at risk of inferior clinical and financial outcomes compared to their non‐ostomy counterparts. We further hypothesized center operative emergency general surgery (EGS) volume to be inversely associated with rates of severe maternal morbidity (SMM) complications.

## 2 METHODS

This was a retrospective cohort study using the National Readmissions Database (NRD). The NRD is an all‐payer administrative database developed by the Healthcare Cost and Utilization Project (HCUP) and includes inpatient discharges from approximately 30 US states, representing roughly 60% of all US hospitalizations and over 60% of the US resident population in recent years. Using validated survey‐weighting methods, the NRD allows for generation of nationally representative estimates for non‐federal, short‐stay hospitalizations across the United States. Readmissions are tracked across hospitals within the same state and a calendar year using verified patient linkage numbers.

All adults (≥18 years) birth hospitalizations entailing vaginal or cesarean delivery were tabulated from 2016 to 2022 National Readmissions Database using previously validated *International Classification of Diseases 10th Revision* (ICD‐10) codes [11] Records involving inter‐hospital transfers or missing key data on age, sex, costs, income or payer status were not considered (Figure S1). The cohort was stratified based on the presence of an ostomy at the time of admission (*Ostomy* vs. *non‐Ostomy*) (Table S1).

Patient and hospital characteristics including age, primary payer, comorbidities, hospital teaching status, bed size, and for‐profit ownership, were defined in accordance with the NRD Data Dictionary [12]. Non‐home discharge was defined as patient disposition to short‐term care or skilled‐nursing facilities. Costs were calculated by applying center‐specific cost to charge ratios to overall hospitalization charges followed by inflation adjustment to the 2022 Personal Health Care Price Index [13]. Separately, the annual caseload of EGS operations at each center was tabulated using validated ICD codes. EGS were defined as non‐elective admissions for large bowel resection, small bowel resection, repair of a perforated ulcer, lysis of peritoneal adhesions, laparotomy, appendectomy, and cholecystectomy [14, 15]. Centers were categorized into low‐, medium‐, and high‐volume tertiles based on the annual number of EGS hospitalizations. Briefly, unique hospital identifiers were used to tabulate institutional caseload of the reporting hospital, generating volume cutoffs at the 33rd and 67th percentiles. Ostomy‐related complications were subcategorized in hemorrhage complications, infectious complications, obstructive complications, other complications, ostomy repair/reversal operations, large bowel resection and small bowel resection.

The primary endpoint of the study was SMM, defined by the [16, 17]. We secondarily considered the individual complication categories, in‐hospital mortality, and markers of resource utilization (inpatient costs, length of stay [LOS], non‐home discharge, and 30‐day non‐elective readmissions). We furthermore considered the development of ostomy complications categorized as: hemorrhage complications (bleeding requiring intervention), infectious complications (such as surgical site infections), obstructive complications (bowel or stoma obstruction), other complications (those not fitting standard categories, including dehydration), ostomy repair/reversal procedures, and both large and small bowel resection procedures that were necessary following delivery (Tables S2–S4). We compared these outcomes between vaginal and cesarean deliveries. Trends across categories were assessed using a nonparametric test for trend (nptren).

All analyses incorporated the NRD survey design, including discharge‐level weights, NRD‐defined strata, and hospital‐level clustering, in accordance with HCUP guidelines. Standard errors were estimated using Taylor‐series linearization. Entropy balancing was used to improve cohort comparability, and elastic net regularization guided covariate selection for multivariable models, with robust standard errors clustered at the hospital level when needed.

Categorical variables are reported as percentages (%) while continuous variables are shown as means with standard deviation (SD) or medians with interquartile range (IQR), if not normally distributed. Bivariate comparisons were performed using the Pearson χ^2^ and Mann‐Whitney *U* tests, as appropriate. The Nelson–Aalen method was used to estimate the cumulative hazards of readmission within 30 days of discharge. Covariate selection for all models was guided by Elastic net regularization, an automated technique which optimizes model parsimony while reducing collinearity among factors [18]. We used entropy balancing as the first‐line statistical approach to mitigate the influence of significant intergroup differences. Briefly, this reweighting algorithm generates a balanced distribution of covariates and has been shown to be superior to propensity matching. Following application of entropy balancing‐derived weights, multivariable logistic and linear regressions were developed to characterize the association of various covariates with in‐hospital mortality and post‐delivery complications [19]. Factors included in risk‐adjusted models incorporated age, primary payer, income quartile, expanded obstetric comorbidity index (EOCI) score, hospital teaching status, hospital bed size, and ostomy indication diagnoses (inflammatory bowel disease, colorectal cancer, diverticular disease, bowel perforation, and other gastrointestinal conditions requiring ostomy). The EOCI is a validated, weighted comorbidity scoring system specifically developed for prediction of severe maternal morbidity in obstetric populations [20]. Linear and logistic model estimates are reported as beta‐coefficients (β) or adjusted odds ratios (AORs), as appropriate, both with 95% confidence intervals (CIs).

All statistical analyses were performed using Stata 16.0 (StataCorp). Due to the limited dataset nature of NRD, this study was deemed exempt from full review by the Institutional Review Board at the University of California, Los Angeles.

## 3 RESULTS

### 3.1 Patient and hospital characteristics

Of an estimated 13,292,764 birth hospitalizations over the study period, 926 (∼0.01%) comprised the *Ostomy* cohort. The prevalence of ostomy during deliveries increased 1.5‐fold during 2016–2022, from 7.1 to 10.6 per 100,000 deliveries overall, with specific increases observed for both cesarean (15.3 to 19.9 per 100,000) and vaginal (3.0 to 7.5 per 100,000) deliveries (Figure S2) (nptrend < 0.001). Throughout the study period, ostomy prevalence remained substantially higher in patients who underwent cesarean compared to vaginal deliveries (Figure S2). Relative to others, *Ostomy* patients were older (31 [27–34] vs. 29 [25–33] years) and demonstrated higher rates of preeclampsia (18.4% vs. 5.7%; *p* < 0.001), cardiac arrhythmias (2.5% vs. 1.0%; *p* < 0.001) and liver disease (1.1% vs. 0.4%; *p* = 0.01). *Ostomy* patients more often cared for at large (69.0% vs. 55.0%; *p* < 0.001), high‐volume EGS hospitals (76.1% vs. 61.6%; *p* < 0.001, Table 1), compared to their *non‐Ostomy* counterparts. Delivery characteristics were not significantly different between *Ostomy* and *non‐Ostomy* groups with the exception of prior cesarean which was more prevalent among *Ostomy* (25.4% vs. 17.9%; *p* < 0.001, Table 1).

**TABLE 1:** Demographic, clinical, and hospital characteristics, stratified by ostomy status.

	Ostomy (*n* = 926)	Non‐ostomy (*n* = 13,291,838)	*p* value
Age (years) (median [Q1–Q3])	31 [27–34]	29 [25–33]	<0.001
Expanded Obstetric Comorbidity Index (median [Q1–Q3])	13 [9–24]	2 [0–11]	<0.001
Income quartile (%)			0.60
75%	23.1	21.5	
51%–75%	24.9	25.5	
26%–50%	26.7	26.3	
0%–25%	25.2	26.7	
Insurance coverage (%)			<0.001
Private	57.3	54.1	
Medicare	7.3	0.8	
Medicaid	31.4	40.6	
Other payer	4.0	4.5	
Comorbidities (%)			
Chronic pulmonary disease	7.5	5.9	0.05
Arrhythmia	2.5	1.0	<0.001
Coagulopathy	3.3	2.4	0.06
Gestational diabetes	8.6	9.7	0.25
Preeclampsia	8.4	5.7	0.01
Liver disease	1.1	0.4	0.01
Hospital teaching status (%)			<0.001
Non‐metropolitan	4.4	9.0	
Metropolitan non‐teaching	10.0	18.8	
Metropolitan teaching	85.6	72.2	
Hospital bed size (%)			<0.001
Small	11.0	17.5	
Medium	19.9	27.5	
Large	69.0	55.0	
Hospital EGS volume (%)			<0.001
Low	6.8	12.8	
Medium	17.1	25.5	
High	76.1	61.6	
Delivery factors (%)			
Dystocia	1.2	1.8	0.23
Prior cesarean	25.4	17.9	<0.001
Medical induction of labor	16.1	19.4	0.01
Premature rupture of membrane	10.2	8.8	0.14
Superimposed preeclampsia	1.4	1.1	0.44

*Note*: Reported as percentages, unless otherwise noted. Statistical significance was set at *α* ≤ 0.05.

Abbreviations: EGS, emergency general surgery; IQR, interquartile range.

### 3.2 Unadjusted outcomes

**FIGURE 1:**
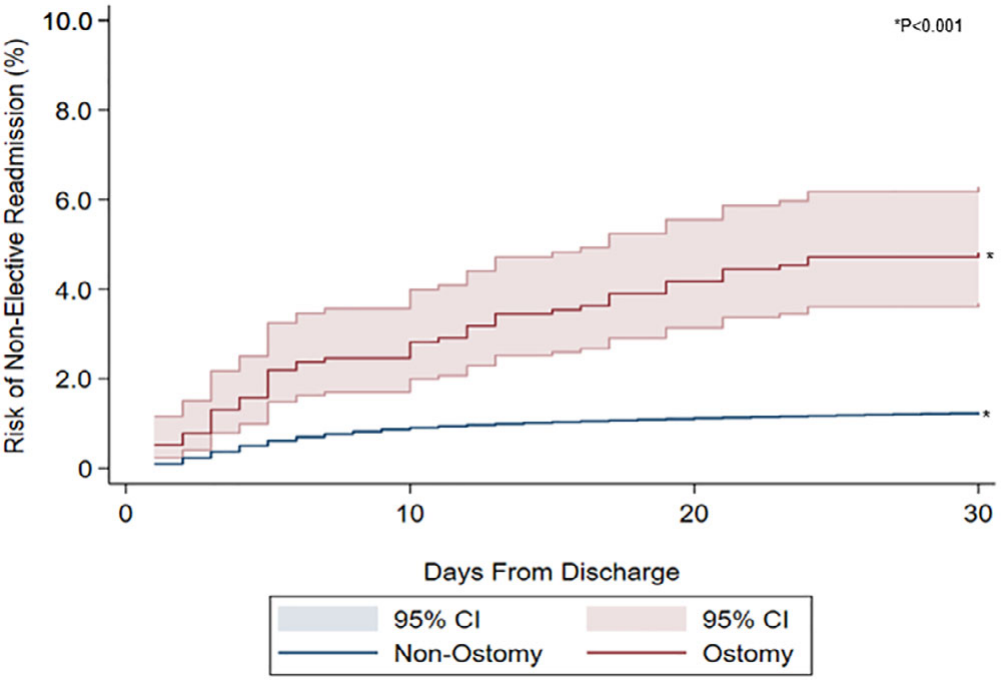
Nelson–Aalen cumulative hazards of non‐elective readmissions within 30 days of discharge stratified by the presence of maternal ostomy. CI: confidence interval. *p < 0.001.

On unadjusted analysis, *Ostomy* patients were more likely to experience SMM (5.2% vs. 3.1%; *p* = 0.01) complications, relative others. Furthermore, *Ostomy* exhibited greater hospitalization costs (7.0k [4.9k–10.5k] vs. $5.0k [3.5k–7.4k]; *p* < 0.001), 30‐day nonelective readmissions (3.7% vs. 1.2%; *p* < 0.001, Figure 1) and inpatient days (3 [2–4] vs. 2 [2–3] days, Table 2), compared to *non‐Ostomy*.

**TABLE 2:** Unadjusted and risk‐adjusted outcomes.

	Ostomy (*n* = 926)	Non‐ostomy (*n* = 13,291,838)	Unadjusted OR/β (CI)	Estimate AOR/β (CI)
Clinical outcomes				
In‐hospital mortality	–	0.02	1.00 (–)	1.00 (–)
SMM	5.2	3.1	1.82 (1.36–2.43)	1.57 (1.04–2.36)
Resource utilization				
Costs (USD $1000s, median [Q1–Q3])	7.0 [4.9–10.5]	5.0 [3.5–7.4]	+2.39 (1.68–3.10)	+1.18 (0.47–1.89)
Length of Stay (Days, median [Q1–Q3])	3 [2–4]	2 [2–3]	+1.01 (0.63–1.39)	+0.41 (0.01–0.81)
30‐Day nonelective readmission	3.7	1.2	2.37 (1.65–3.41)	1.54 (0.91–2.60)
Non‐home discharge	–	0.1	2.19 (0.50–9.59)	0.15 (0.06–0.36)

*Note*: Outcomes reported as percentages or medians with IQR. Logistic model outputs are reported as adjusted odds ratios (AOR) with 95% confidence intervals (95% CIs).

Abbreviations: β, adjusted incremental difference; CI, confidence interval; IQR, interquartile range, USD, United States dollars.

### 3.3 Risk‐adjusted outcomes

**FIGURE 2:**
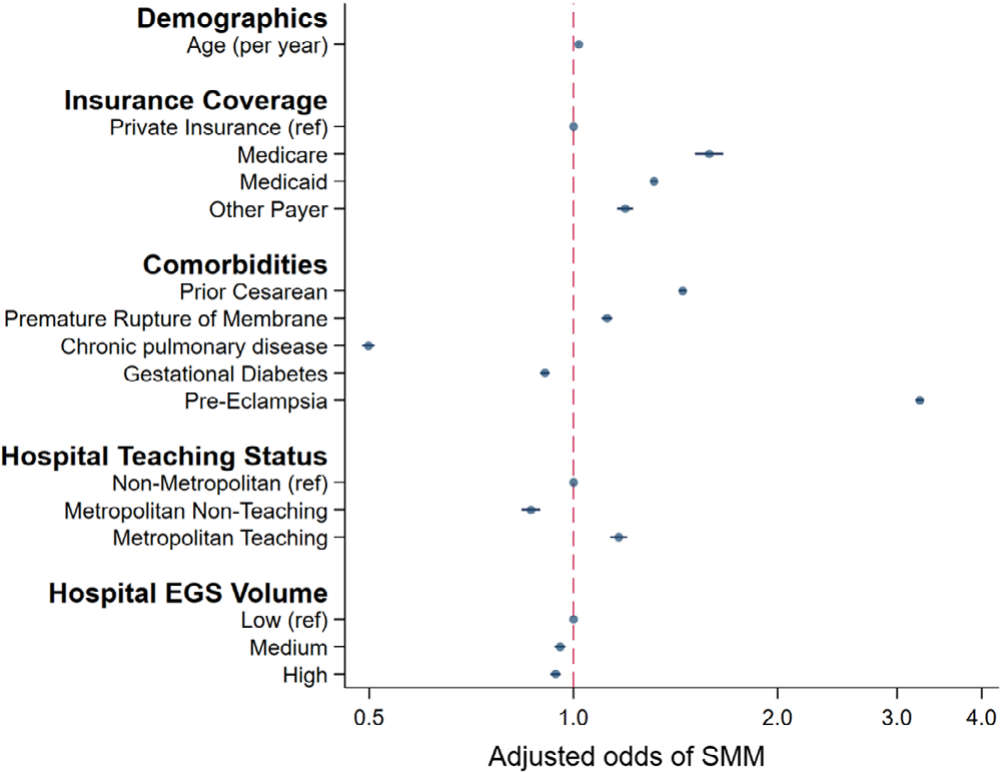
Demographic and hospital‐level factors associated with SMM in delivery patients. *X*‐axis is shown on a logarithmic scale EGS, emergency general surgery.

Adequate balance of patient characteristics and comorbidities was achieved between cohorts using entropy balancing. Following comprehensive risk adjustment, the presence of an ostomy was linked with increased odds of SMM (AOR, 1.57; 95% CI, 1.04–2.36). Notably, treatment at medium (AOR, 0.95; 95% CI, 0.94–0.97) and high‐volume EGS centers (AOR, 0.94; 95% CI, 0.92–0.96) was associated with reduced odds of SMM as shown in Figure 2. Moreover, ostomy was associated with increased hospitalization costs by $1.18k (95% CI, 473–1890), shorter length of stay by 0.41 days (95% CI, 0.01–0.81) and increased odds of 30‐day readmission (AOR, 1.54; 95% CI, 0.91–2.60, Table 2). Risk‐adjusted analysis comparing cesarean versus vaginal delivery among ostomy patients revealed no significant differences in ostomy‐related complications (Table 3).

**TABLE 3:** Risk‐adjusted odds of ostomy‐related operation associated with cesarean delivery (ref: vaginal delivery).

	Estimate (AOR/β)	95% CI
Ostomy‐related complications		
Hemorrhage complications	1.00	–
Infectious complications	0.94	0.83–1.05
Other complications	1.01	0.93–1.10
Obstructive complications	0.95	0.85–1.07
Ostomy repair/reversal	0.98	0.88–1.09
Large bowel resection	1.00	–
Small bowel resection	1.07	0.88–1.30

*Note*: Logistic model outputs are reported as adjusted odds ratios (AOR) with 95% confidence intervals (95% CIs).

Abbreviation: β, adjusted incremental difference; CI, confidence interval.

## 4 DISCUSSION

With the increasing prevalence of conditions requiring ostomy in younger adults, the number of women with ostomies considering pregnancy is growing. In the present national study, we investigated the association of maternal ostomy and clinical outcomes during birthing hospitalizations and made several important observations. While the prevalence of patients with ostomy undergoing delivery has increased significantly from 2016 to 2022, such patients face increased costs, length of stay and risk of short‐term rehospitalizations. Notably, treatment at medium and high‐volume EGS centers appears to mitigate some of the adverse consequences of ostomy. Several of these findings warrant further discussion.

The prevalence of ostomy during deliveries increased 1.5‐fold during the 7‐year study period from 2016 to 2022, with rates in cesarean deliveries 2.7 to 4 times higher than those observed in vaginal deliveries. While this temporal pattern may reflect a growing number of reproductive‐age individuals living with ostomies due to improved long‐term disease management and survivorship, it may also be influenced by evolving administrative coding practices and more complete capture of ostomy status over time. Our findings are in agreement with Whitley et al. who reported ostomy formation to effectively manage symptoms of Inflammatory Bowel Disease (IBD) and familial adenomatous polyposis, allowing women to achieve a sense of readiness for conception, pregnancy, and motherhood [9]. Additionally, the higher prevalence of ostomy among cesarean compared to vaginal highlights the clinical tendency toward operative delivery in this population which is consistent with the findings of Kelly et al who found cesarean rate of 90% for IBD patients with stoma, reflecting complexity and thus surgical preference in managing these cases [6].

We noted markers of expenditures including hospitalization costs, length of hospitalization, and 30‐day readmission rates to be significantly higher among ostomy patients. In the present work, presence of an ostomy was associated with a ∼ $1.38k increment in hospitalization costs which could be due to increased interventions in this population. Similar to our findings, Carlsson et al. reported healthcare costs for ostomy patients to remain elevated even a decade after the initial operation [21]. Moreover, we noted 30‐day readmission rates for ostomy patients to be more than double that of their non‐ostomy counterparts, suggesting the presence of complications that require additional care after discharge. This is consistent with the work of Fish et al. who reported 28% of patients with new ileostomies to be readmitted within 60 days [22]. As highlighted by Sanaiha et al. and Tyler et al., the economic impact of ostomies extends beyond the immediate hospitalization while the need of potential long‐term support contributes significantly to additional healthcare costs [23, 24].

Among ostomy patients experiencing childbirth, treatment at medium‐ and high‐volume EGS centers were found to be associated with reduced odds of SMM. This finding may reflect the availability of institutional resources, multidisciplinary infrastructure, and general surgery expertise at centers that routinely manage complex abdominal pathology. Works of Mehta et al. and Ogoila et al. have previously demonstrated a positive volume outcome relationship in EGS with high‐volume hospitals yielding lower rates of mortality and improved outcomes attributable to surgeon experience and institutional resources [25, 26]. Our work extends the above findings by demonstrating a positive cross‐volume association between EGS expertise and delivery outcomes. We proffer the presence of specialized care and infrastructure at high‐volume EGS center to at least, in part, contribute to this observed relationship. Taken together, our work suggests that pregnant patients with ostomy may be better served at institutions with EGS expertise and should ideally receive their peripartum care at such facilities.

This work highlights the importance of preconception counseling and assessing pregnancy intention in the optimal care for women with ostomies, as they can significantly impact outcomes. This population faces unique challenges during pregnancy, including increased risks of severe maternal morbidity, higher rates of cesarean delivery, and stoma‐related complications. Preconception counseling allows healthcare providers to optimize medical management, address potential risks, and ensure that women are fully informed about the implications of pregnancy with an ostomy. By incorporating these steps into care, clinicians can better support women with ostomies in achieving safer pregnancies and improved outcomes [27].

Our study has several important limitations. Due to the retrospective nature of the NRD and reliance on ICD coding as well as variations in reporting practices between clinicians, hospitals, and regions, our findings may subject to unknown confounders. Similarly, patient preferences and operative decision‐making could not be ascertained. Additionally, we did not consider the information regarding the indication for ostomy creation or its duration prior to pregnancy, which limits conclusions regarding specific types of ostomies and their individual impact. Importantly, the observed temporal increase in ostomy prevalence among delivery hospitalizations may not represent a true rise in pregnancy rates among patients with ostomy. Instead, it may reflect improvements in coding practices and documentation over time, including broader electronic medical record adoption and more consistent capture of ostomy status on administrative claims. Additionally, although we observed lower odds of severe maternal morbidity at medium‐ and high‐volume EGS centers, this association may be confounded by referral patterns and delivery circumstances. For example, patients presenting in urgent or emergent situations may be more likely to deliver at hospitals without high EGS availability, and NRD lacks granular clinical details (e.g., disease severity, timing of ostomy creation, surgical indication, planned vs. unplanned delivery, or availability of on‐call surgical consultation) that could influence both site of care and outcomes. Therefore, our findings should be interpreted as associative rather than causal. Despite these limitations, we used a large, all‐payer database, and robust statistical methods to reduce the risk of bias and increase the generalizability of our findings at the national level.

In conclusion, we noted maternal ostomy to be associated with increased severe maternal morbidity, higher resource utilization, and greater readmission rates in birthing hospitalizations. Further, we found treatment at medium and high‐volume EGS centers to be associated with reduced odds of severe maternal morbidity, highlighting the importance of specialized care for this high‐risk population. These findings call for future work investigating optimal management strategies and the development of specialized protocols for pregnant women with ostomies which ensure optimization of maternal outcomes while minimizing complications.

## AUTHOR CONTRIBUTIONS

*Conceptualization*: Yalda Afshar. *Methodology*: Sona Mahrokhi, Amulya Vadlakonda, and Peyman Benharash. *Software*: Amulya Vadlakonda, Troy Coaston, and Melissa Justo. *Data Curation*: Sona Mahrokhi and Amulya Vadlakonda. *Investigation*: Konmal Ali. *Validation*: Troy Coaston and Melissa Justo. *Formal Analysis*: Sona Mahrokhi. *Supervision*: Yalda Afshar and Peyman Benharash. *Funding Acquisition*: Peyman Benharash. *Visualization*: Troy Coaston and Gizem Bilgili. *Project Administration*: Amulya Vadlakonda, Troy Coaston and Melissa Justo. *Resources*: Sona Mahrokhi and Amulya Vadlakonda. *Writing—Original Draft*: Sona Mahrokhi and Amulya Vadlakonda. *Writing—Review & Editing*: Amulya Vadlakonda, Yalda Afshar and Peyman Benharash.

## CONFLICT OF INTEREST STATEMENT

P.B. reports receiving proctoring fees from AtriCure. This manuscript does not discuss any related products or services. Y.A. serves as a consultant for Mirvie and Bio‐Rad, which is not relevant to this work. All other authors declare no conflicts of interest.

## FUNDING INFORMATION

The authors received no specific funding for this work.

## Supporting information



Supporting Information

Supporting Information

Supporting Information
