# Supplementary material for: Maternal outcomes among ostomy patients undergoing vaginal and cesarean deliveries: A national analysis
Source: Pregnancy (Hoboken). 2026 Feb 12;2(2):e70261. doi: 10.1002/pmf2.70261 (PMC13344338; doi:10.1002/pmf2.70261)
Supplement: Supplementary file 1 [file PMF2-2-e70261-s003.docx]

| **Supplementary Table 2: Ostomy-Related Complications** | | |
| --- | --- | --- |
| **Outcome category** | **Variable name in code** | **ICD-10-CM code patterns used (from your code)** |
| **Ostomy complications (colostomy-specific)** | colcomp | **K94.00, K94.01, K94.02, K94.03, K94.09** |
| **Ostomy complications (ileostomy-specific)** | ileocomp | **K94.10, K94.11, K94.12, K94.13, K94.19** |
| **Hemorrhage complications** | hemorcomp | **K94.01, K94.11** |
| **Infectious complications** | infcomp | **K94.02, K94.12**, **A49.01, A49.02, L08.89, K68.11, T81.40–T81.49, L08.9, A49.1, A49.8, A49.9, B37.9, L08.0, N39.0, L88** |
| **Obstructive complications** | obscomp | **K43.3, K43.5, K56.3, K31.5** |
| **Other complications** | othercomp | **K94.00, K94.03, K94.09, K94.10, K94.13, K94.19, L98.49, L24.5, L24.9, L92.8, E86.0** |
| **Skin complications (broad capture)** | skincomp | **A49.01, A49.02, L08.89, L98.49, L08.9, A49.1, A49.8, A49.9, B37.9, L08.0, L24.5, L24.9, L92.8, L88*** |

| **Supplementary Table 1**: Ostomy Status at Index Delivery Admission | |
| --- | --- |
| **Variable** | ICD-10 Code |
| **Colostomy** | Z93.3, Z43.3 |
| **Ileostomy** | Z93.2, Z43.2 |

| **Supplementary Table 3: Ostomy Repair/Reversal Procedures** | | |
| --- | --- | --- |
| **Outcome** | **Variable name in code** | **ICD-10-PCS codes used** |
| **Ileostomy repair/revision procedures** | ileorepair | 0DQ80ZZ, 0DQ83ZZ, 0DQ84ZZ, 0DQ87ZZ, 0DQ88ZZ, 0DQ90ZZ, 0DQ93ZZ, 0DQ94ZZ, 0DQ97ZZ, 0DQ98ZZ, 0DQA0ZZ, 0DQA3ZZ, 0DQA4ZZ, 0DQA7ZZ, 0DQA8ZZ, 0DQB0ZZ, 0DQB3ZZ, 0DQB4ZZ, 0DQB7ZZ, 0DQB8ZZ |
| **Colostomy repair/reversal procedures** | colrepair | 0DQN0ZZ, 0DQM0ZZ, 0DQL0ZZ, 0DQG0ZZ, 0DQP0ZZ, 0DQK0ZZ, 0DQH0ZZ, 0DQF0ZZ, 0DQE0ZZ, 0DQN4ZZ, 0DQM4ZZ, 0DQL4ZZ, 0DQG4ZZ, 0DQP4ZZ, 0DQK4ZZ, 0DQH4ZZ, 0DQF4ZZ, 0DQE4ZZ, 8E0W4CZ, 8E0W8CZ |
| **Any ostomy repair/reversal** | ostomyrepair | Any code listed above for ileorepair **or** colrepair |

| **Supplementary Table 4: Bowel Resections Following Delivery** | | |
| --- | --- | --- |
| **Outcome** | **Variable name in code** | **ICD-10-PCS code used** |
| **Large bowel resection (LBR)** | lbr | 0DBE0, 0DBE3, 0DBE4, 0DBF0, 0DBF3, 0DBF4, 0DBG0, 0DBG3, 0DBG4, 0DBH0, 0DBH3, 0DBH4, 0DBK0, 0DBK3, 0DBK4, 0DBL0, 0DBL3, 0DBL4, 0DBM0, 0DBM3, 0DBM4, 0DBN0, 0DBN3, 0DBN4, 0DBP0, 0DBP3, 0DBP4, 0DBQ0, 0DBQ3, 0DBQ4, 0DBC0, 0DBC3, 0DBC4, 0DTE0, 0DTE4, 0DTF0, 0DTF4, 0DTG0, 0DTG4, 0DTH0, 0DTH4, 0DTK0, 0DTK4, 0DTL0, 0DTL4, 0DTM0, 0DTM4, 0DTN0, 0DTN4, 0DTP0, 0DTP4, 0DTQ0, 0DTQ4, 0DTC0, 0DTC4 |
| **Small bowel resection (SBR)** | sbr | 0DB80, 0DB83, 0DB84, 0DB90, 0DB93, 0DB94, 0DBA0, 0DBA3, 0DBA4, 0DBB0, 0DBB3, 0DBB4, 0DT80, 0DT84, 0DT90, 0DT94, 0DTA0, 0DTA4, 0DTB0, 0DTB4 |

| **Supplementary Table 5**. ICD-10-CM/PCS coding definitions for SMM components (CDC-based)  Definitions were based on CDC SMM ICD-10-CM/PCS criteria; codes are shown in programming format for NRD. | |
| --- | --- |
| **Acute Myocardial Infarction** | "I21", "I22" |
| **Aneurysm** | "I71", "I790" |
| **Acute Renal Failure** | "N17", "O904" |
| **Acute Respiratory Distress Syndrome** | "J80", "J951", "J952", "J953", "J960", "J962", "J969", "R092", "J9582", "R0603" |
| **Amniotic Fluid Embolism** | "O8812", "O8813", "O88112", "O88113","O88119” |
| **Cardiac Arrest or Ventricular Fibrillation** | "I46", "I490" |
| **Conversion of Cardiac Rhythm** | "5A12012", "5A2204Z" |
| **Disseminated Intravascular Coagulation** | "D688", "D689", "O670", "O723","D65”,"O45002", "O45003", "O45009", "O45012", "O45013", "O45019", "O45022","O45023", "O45029", "O45092", "O45093", "O45099", "O46002", "O46003","O46009", "O46012", "O46013", "O46019", "O46022", "O46023", "O46029","O46092", "O46093", "O46099" |
| **Blood Transfusions** | "30230H0", "30230K0", "30230L0", "30230M0", "30230N0", "30230P0" ,"30230R0", "30230T0", "30230H1", "30230K1", "30230L1", "30230M1" ,"30230N1", "30230P1", "30230R1", "30230T1", "30233H0", "30233K0" ,"30233L0", "30233M0", "30233N0", "30233P0", "30233R0", "30233T0" ,"30233H1", "30233K1", "30233L1", "30233M1", "30233N1", "30233P1" ,"30233R1", "30233T1", "30240H0", "30240K0", "30240L0", "30240M0" ,"30240N0", "30240P0", "30240R0", "30240T0", "30240H1", "30240K1" ,"30240L1", "30240M1", "30240N1", "30240P1", "30240R1", "30240T1" ,"30243R0", "30243T0", "30243H1", "30243K1", "30243L1", "30243M1" ,"30243H0", "30243K0", "30243L0", "30243M0", "30243N0", "30243P0" ,"30243N1", "30243P1", "30243R1", "30243T1" |
| **Eclampsia** | "O15" |
| **Heart Failure or Arrest during Procedure or Surgery** | "I97120", "I97121", "I97130", "I97131", "I97710", "I97711" |
| **Puerperal Cerebrovascular Disorders** | "G45", "G46", "I60", "I61", "I62", "I65", "I66", "I67", "I68","A812", "I631", "I632", "I633", "I634", "I635", "I636", "I638", "I639", "H340", "O873","G9349", "I6300", "I6301", "O2250","O2252","O2253","I97810", "I97811", "I97820", "I97821" |
| **Acute Pulmonary Edema** | "I501", "I509", "J810", "I5020", "I5021", "I5023", "I5030", "I5031", "I5033", "I5040", "I5041", "I5043", "I5082", "I5083", "I5084", "I5089", "I50810", "I50811", "I50813", "I50814” |
| **Severe Anesthesia Complications** | "O740", "O741", "O742", "O743", "O890", "O891", "O892", "O29292", "O29293", "O29294", "O29295", "O29296", "O29297", "O29298", "O29299""O29212", "O29213", "O29214", "O29215", "O29216", "O29217", "O29218", "O29219""O29192", "O29193", "O29194", "O29195", "O29196", "O29197", "O29198", "O29199""O29122", "O29123", "O29124", "O29125", "O29126", "O29127", "O29128", "O29129""O29112", "O29113", "O29114", "O29115", "O29116", "O29117", "O29118", "O29119""T882XXA", "T883XXA" |
| **Sepsis** | "A327""A40", "A41", "I76", "O85""O8604", "R6520", "R6521""T8112XA, T8144XA" |
| **Shock** | "R57""O751""T782XXA", "T8110XA", "T8111XA", "T8119XA", "T886XXA" |
| **Sickle Cell Disease with Crisis** | "D5700", "D5701", "D5702""D57211", "D57212", "D57219", "D57411", "D57412", "D57419", "D57811", "D57812", "D57819" |
| **Air and Thrombotic Embolism** | "I26", "O88""T800XXA" |
| **Hysterectomy** | "0UT90ZL", "0UT90ZZ", "0UT97ZL", "0UT97ZZ" |
| **Temporary Tracheostomy** | "0B110F4", "0B113F4", "0B114F4" |
| **Mechanical Ventilation** | "5A1935Z", "5A1945Z", "5A1955Z" |
